# Oxidative stress causes hypertension and activation of nuclear factor-κB after high-fructose and salt treatments

**DOI:** 10.1038/srep46051

**Published:** 2017-04-11

**Authors:** Waleska C. Dornas, Leonardo M. Cardoso, Maísa Silva, Natália L. S. Machado, Deoclécio A. Chianca-Jr., Andréia C. Alzamora, Wanderson G. Lima, Vincent Lagente, Marcelo E. Silva

**Affiliations:** 1 Research in Biological Sciences - NUPEB, Universidade Federal de Ouro Preto, Minas Gerais, Brasil; 2 UMR991, INSERM, Université de Rennes 1, Rennes, France; 3 Department of Biological Sciences, Instituto de Ciências Exatas e Biológicas, Universidade Federal de Ouro Preto, Minas Gerais, Brasil; 4 Department of Foods, Escola de Nutrição, Universidade Federal de Ouro Preto, Minas Gerais, Brasil

## Abstract

There is evidence that diets rich in salt or simple sugars as fructose are associated with abnormalities in blood pressure regulation. However, the mechanisms underlying pathogenesis of salt- and fructose-induced kidney damage and/or consequent hypertension yet remain largely unexplored. Here, we tested the role of oxidative state as an essential factor along with high salt and fructose treatment in causing hypertension. Fischer male rats were supplemented with a high-fructose diet (20% in water) for 20 weeks and maintained on high-salt diet (8%) associate in the last 10 weeks. Fructose-fed rats exhibited a salt-dependent hypertension accompanied by decrease in renal superoxide dismutase activity, which is the first footprint of antioxidant inactivation by reactive oxygen species (ROS). Metabolic changes and the hypertensive effect of the combined fructose-salt diet (20 weeks) were markedly reversed by a superoxide scavenger, Tempol (10 mg/kg, gavage); moreover, Tempol (50 mM) potentially reduced ROS production and abolished nuclear factor-kappa B (NF-κB) activation in human embryonic kidney HEK293 cells incubated with L-fructose (30 mM) and NaCl (500 mosmol/kg added). Taken together, our data suggested a possible role of oxygen radicals and ROS-induced activation of NF-κB in the fructose- and salt-induced hypertension associated with the progression of the renal disease.

It is widely accepted and seems irrefutable that metabolic stress is linked to insulin resistance (IR) with cardiovascular implications of oxidative stress in insulin resistant conditions^1^,^2^,^3^. Previous studies have demonstrated that this association is not only restricted to IR in type 2 diabetes, but is also evident in the metabolic syndrome (MS) with oxidative stress related to hyperglycemia, hyperinsulinemia, hypertriglyceridemia, and inadequate antioxidant defenses^4^,^5^. We had recently demonstrated that IR increases in fructose-fed rats on a high-salt diet^6^ with uncontrolled free radical production might be one of the mechanisms underlying the development of co-morbidities in MS. Particularly, increasing evidence suggests that nutritional disorder is a leading cause of diabetes and cardiovascular diseases with enhanced production of reactive oxygen species (ROS) contributing to dysregulation of physiological processes. Oudot *et al*.^7^, for example, showed a co-existence of oxidative stress in the insulin-resistant states with both added salt and sugars presenting an effect on blood pressure (BP) and a beneficial influence of dietary sodium restriction on attenuation of the pro-oxidant effect due to fructose treatment. Nevertheless, a direct evidence for the antioxidant-induced protection against cell and tissue injuries in hypertension has not been well established.

Interestingly, extracellular superoxide dismutase (SOD) is one of the main enzymatic antioxidants that scavenging superoxide radicals (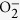), and various reports have revealed that a membrane-permeable, metal-independent SOD mimetic can reduce BP in experimental models of hypertension^8^,^9^,^10^,^11^. Accordingly, protective effect of SOD decreasing oxidative stress conditions is believed to be involved in hypertension. Specifically, it should be noted that ROS may act as signal transduction messengers for several important transcription factors, such as nuclear factor-kappa B (NF-κB)^12^,^13^, although this can be prevented by antioxidants^14^. NF-κB is a transcription protein that upregulates many genes involved in inflammation. On activation by extracellular stimuli, NF-κB translocates from the cytoplasm into the nucleus and regulates specific gene expression that possibly may be attributable to elevated ROS production. Indeed, there is increasing evidence suggesting a strong relationship between hypertension and inflammation, as well as end-organ damage due oxidative stress, where an imbalance between the production of ROS and antioxidants leading to oxidative stress because of either increased ROS generation, or a depressed antioxidant system, or both, is believed to be involved in the progression of renal disease/hypertension^15^.

The current study was designed to elucidate the contribution of ROS in the hypertensive response to an established model with fructose-fed rats maintained on high-salt load. Our results revealed a role for oxygen radicals in the maintenance of hypertension induced by high fructose-salt with a possible role of ROS-NF-κB activation.

## Results

### Effect of high-salt and fructose on body weight, food and fluid consumption, and glucose tolerance

**Figure 1:**
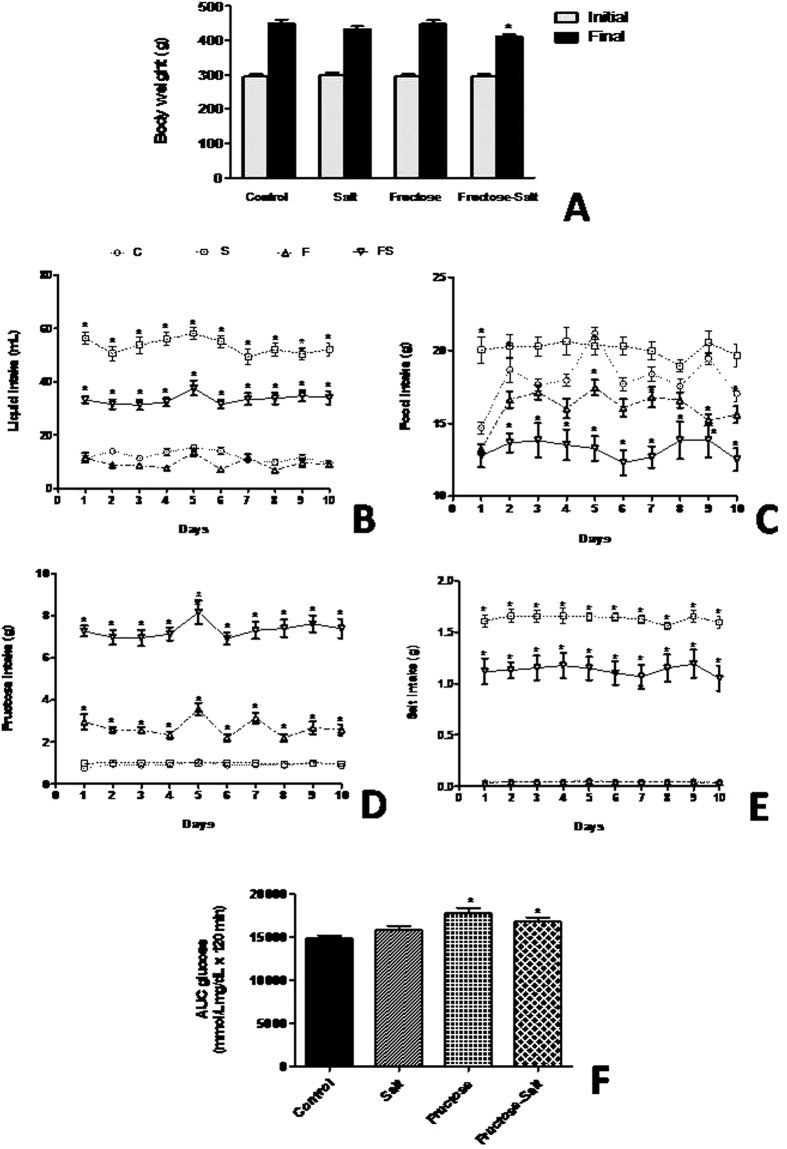
Characteristic of fructose-fed rats on high-salt diet. Change in body weight (**A**), liquid intake (**B**) food intake (**C**), fructose intake (**D**), salt intake (**E**), and glucose AUC (**F**). Data are presented as means ± SEM. *P < 0.05 compared with control group. n = 10–12 in each group. C, control group; S, high-salt group; F, high fructose group; FS high fructose and high salt after a previous 10 weeks treatment with F.

Throughout our studies, fructose-fed rats on high-salt diet were evaluated and physiologic parameters are shown in the Fig. 1. While initial body weight of the animals among the groups were not significantly different, analysis of variance revealed a significant effect of salt intake on body weight, with high-fructose and salt (FS) animals weighing less (*P* < *0.05*) at the end of 20 weeks than those on the control diet (Fig. 1A). We observed, as expected, that high-salt (S) diet increased (*P* < *0.0001*) water intake compared with the control (C) group, whereas sugar-drinking animals ingested more liquid (*P* < *0.001*) when maintained on a high salt diet (Fig. 1B). In contrast, the high-fructose(F) and FS group showed a reduction (*P* < *0.0001*) in food consumption of approximately 11% and 28% (respectively) due to the greater amount of energy provided by fructose in drinking water (Fig. 1C). This comparison was accomplished by calculating the intake of salt and fructose on a daily basis and then adjusting the salt concentration in food and the fructose concentration in water in the group that received both salt and fructose. FS rats showed greater intake (*P* < *0.0001*) of fructose compared with F rats (Fig. 1D), which also affected salt consumption, as evidenced by lower (*P* < *0.0001*) salt intake in FS rats compared with S rats (Fig. 1E). To determine if high-fructose impair glucose tolerance we evaluate the glucose AUC after an oral glucose tolerance test. Of note, fructose-fed rats increased (*P* < *0.05*) glucose levels over time compared to controls, which suggest impaired insulin action (Fig. 1F).

### Fructose-fed rats increased blood pressure only in group treated with salt load

**Figure 2:**
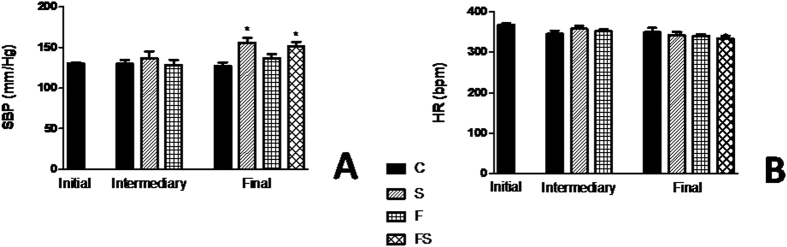
Blood pressure change of fructose-fed rats on high-salt diet. Systolic blood pressure (**A**) and heart rate (**B**). Values are presented as the average of 10 measurements that were recorded continuously throughout the experimental protocol. Data are presented as means ± SEM. *P < 0.05 compared with control group. n = 10–12 in each group. HR, heart rate; SBP, systolic blood pressure; C, control group; S, high-salt group; F, high fructose group; FS, high fructose and high salt group after a previous 10 weeks treatment with F.

To evaluate if fructose and/or salt increases BP, tail plethysmography was performed to measure BP in control, F, S, FS, animals. Figure 2A and B shows the changes BP and heart rate (HR), respectively. Systolic BP (SBP). measured in the conscious rats progressively increased in the high-salt-alone group, and its value on wk 18 was 155 ± 6 mmHg, displaying a significantly increased (*P* < *0.001*) compared with control group. In contrast, although fructose enrichment by itself did not result in increased BP, there was a significant increase in rats when they ate the diet containing fructose and salt (*P* < *0.05*). It should be noted that the increase in BP in response to the FS rats was paralleled by an approximately similar increase in S group, albeit with lower salt intake in time and amount. HR differences among the four dietary groups were not statistically significant.

### Decreased renal SOD activity of fructose-fed rats on high-salt diet

**Figure 3:**
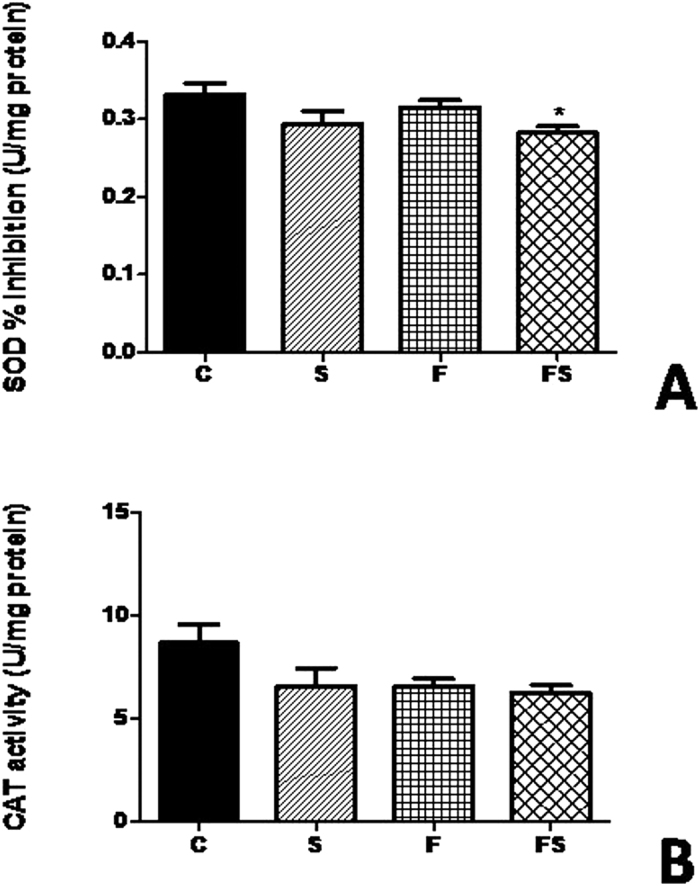
Antioxidant enzymes in the kidney of fructose-fed rats on high-salt diet. SOD activity (**A**) and CAT activity (**B**). Data are presented as means ± SEM. *P < 0.05 compared with control group. n = 10–12 in each group. CAT, catalase; SOD, superoxide dismutase; C, control group; S, high-salt group; F, high fructose group; FS, high fructose and high salt group after a previous 10 weekstreatment with F.

Enzymatic activity of SOD and catalase (CAT) in the kidney from experimental group are showed in the Fig. 3A and B. SOD activity was significantly lower (*P* < *0.005*) in group treated with fructose and salt load, suggesting a depressed antioxidant system in our model. Furthermore, a decrease in the activity of CAT was observed in the fructose and salt groups compared with control, but this did not reach statistical significance.

### Tempol inhibits hypertension and regulates blood parameters

**Figure 4:**
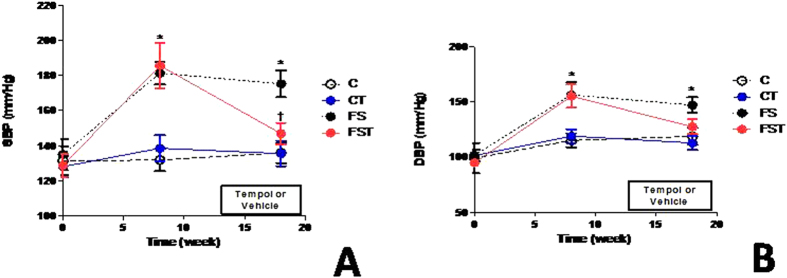
Effect of Tempol on blood pressure of fructose-fed rats on high-salt diet. Systolic blood pressure (**A**), and diastolic blood pressure (**B**). Values are presented as the means ± SEM of BP measurement before (baseline), after 8 weeks of diet, and after Tempol. *P < 0.05 vs. control group and ^†^P < 0.05 vs. FS group at the same experimental time point. n = 10–12 in each group. DBP, diastolic blood pressure; SBP, systolic blood pressure; C, control group; CT, control group + Tempol; FS, high fructose and salt group; FST, high fructose and salt group + Tempol.

Consistent with results in the first set of experiments, FS animals showed elevation (*P* < *0.001*) in BP. Tempol decreased SBP levels (−28 mmHg, *P* < *0.05*) and diastolic BP (DBP). to values similar to C and CT groups (Fig. 4). HR did not differ significantly among treatments during the study (data not shown). Blood glucose, triacylglycerol, urea, creatinine, and uric acid levels were all significantly higher (*P* < *0.05*) in FS animals compared with control animals, but Tempol treatment normalized these parameters (Table 1) to near control levels in FST animals. A decrease in total cholesterol and high density lipoprotein (HDL). (38%, and 48%, *P* < *0.0001*) was observed in animals treated with fructose and salt *versus* control diet. In contrast, Tempol-treated rats on high-salt and fructose diet had the increase levels of serum total cholesterol (1.77 ± 0.2 *vs.*2.1 ± 0.4 mmol/L, *P* < *0.05*) and of HDL (1.1 ± 0.04 *vs*. 1.6 ± 0.1 mmol/L, *P* < *0.0001*) compared to rats without Tempol treatment, although levels were different from those observed in the control (Table 1). The changes in haematocrit increased (*P* < 0.001) by approximately 40% in the FS group and Tempol treatment did not influenced this change. There were no significant changes in sodium and potassium concentrations in the serum (Table 1).

### Tempol change blood profile of lymphocyte and monocyte

**Figure 5:**
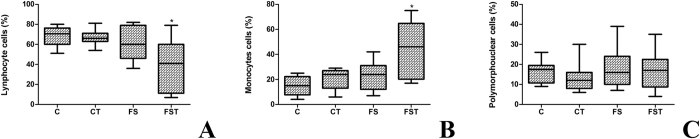
Differential count of leucocytes of fructose-fed rats on high-salt diet and Tempol treatment. Lymphocytes cells **(A**), monocytes cells (**B**), and polymorphonuclear cells (**C**). Boxes with the central mark indicate the median, the edges of the box are the percentiles. *P < 0.05 compared with control group. n = 10–12 in each group. PMN; polypolymorphonuclear; C, control group; S, high-salt group; F, high fructose group; FS high fructose and salt group.

The complete blood count of the fructose-fed rats on high-salt diet and Tempol treatment is presented in the Figure 5. Given the multiorgan nature of these insults, the leukocyte counts were performed and we observed a significantly (*P* < *0.05*) decrease in the of FS group Tempol treated to lymphocyte profile. Particularly, significant (*P* < *0.05*) increases were noted in the monocyte profile when Tempol was utilized compared with those in the control group. There was no significant difference in polymorphonuclear cells (neutrophil, eosinophil and basophil counts between groups).

### No change in SOD expression after fructose-salt treatment in the HEK293 cells

**Figure 6:**
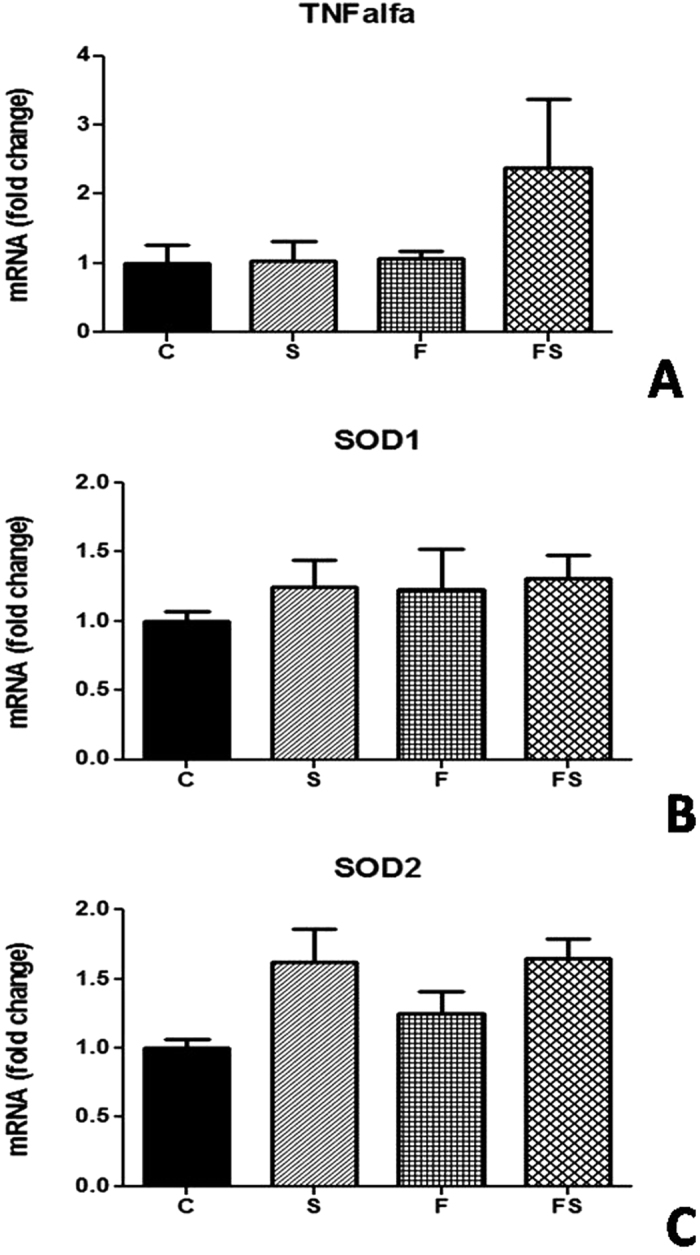
Expression of gene in HEK293 cells treated with fructose, saltfor 4 h. Renal levels of mRNA of TNF-α (**A),** SOD1 (**B**), and SOD2 (**C**). Levels of mRNA were determined by for real-time quantitative RTPCR as described in Methods. n = 6 in each group. TNF-α; tumor necrosis factor alpha; SOD, superoxide dismutase; C, control group; CT, control group + Tempol; FS, high fructose and salt group.

The renal cells did not change in either the fructose and salt only or in combination to TNF-α, SOD1 and SOD2 expression (Fig. 6).

### Tempol blockade ROS production and NF-κB activation in the HEK293 cells

**Figure 7:**
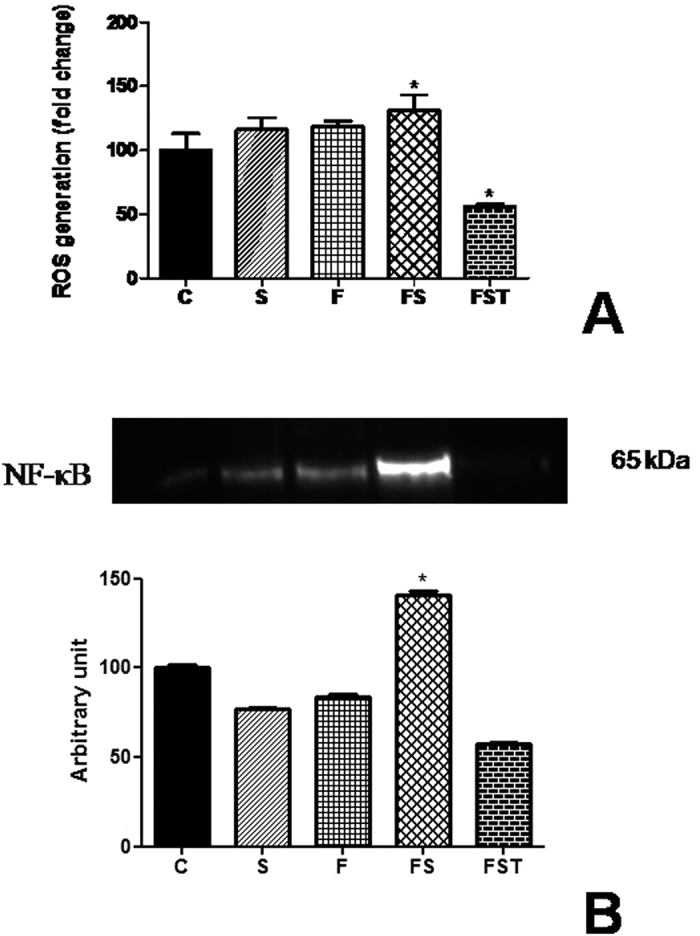
Generation of ROS and expression of NFκB p65 in HEK293 cells treated with fructose, salt, and Tempol for 4 h. Reactive oxygen species production (**A**) and NFκB p65 protein expression (**B**). ROS were quantified by the H2-DCFDA assay. All results are expressed relative to the levels found in control cells, arbitrarily set at a value of 100%. Panel shows bands of NFκB p65 analyzed using Western blotting and quantitative results of detected proteins determined by densitometric analysis. *P < 0.05 compared with control group. n = 3 in each group. ROS, reactive oxygen species; C, control group; CT, control group + Tempol; FS, high fructose and salt group; FST, high fructose and salt group + Tempol.

ROS increased (*P* < *0.001*) in the cells treated with fructose and salt associates (Fig. 7A) and following combined exposure with the free radical scavenger Tempol (50 mM) ROS activation was inhibited (*P* < 0.001) to 50×. Furthermore, we investigated the activation of the transcription factors NF-kB, which has been considered of importance in the progression of chronic renal disease. Immunoblotting showed that NF-kB subunit p65 was increased only when exposed to fructose and salt together in HEK293 cells, whereas Tempol markedly reduced p65 expression (Fig. 7B).

## Discussion

In this study, while our results showed that Fischer rats were salt-sensitive, where salt sensitivity usually reflects changes in BP after a change of dietary sodium intake, BP did not increased in fructose-fed rats. This suggests that rats fed with sugar-rich diets showed metabolic perturbation insufficient to cause hypertension. In addition, we provided functional data to support involvement of oxidative stress to fructose-induced hypertension when salt load is associated. We observed an increase in BP induced by the fructose-salt diet in relation S. group with the same time of treatment using S. diet that contained 8% NaCl, which demonstrated that the ability of a fructose enriched diet to increase BP is modulate by changes in salt intake. In salt-sensitive animals models, fructose-induced hypertensive response with S. diet significantly increased fluid intake^16^, and leads to further oxidative stress^17^. Moreover, we assumed that a superoxide scavenger decreasing renal ROS production may be responsible for intracellular regulation of NF-κB pathway to high fructose-salt treatments; and therefore it seems reasonable to postulate that a high salt intake enhanced the magnitude of fructose-induced BP elevation, with 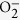 production being an accounted for NF-κB modulation by impaired cardiovascular homeostasis observed in the model used.

The volume of extracellular bodily fluids, including the circulating fluid, tends to increase when a high-salt diet is used; elevating sodium excretion may maintain sodium balance at the expense of increased BP levels^18^. Thirst was stimulated by increased salt ingestion, resulting in intake of more fluid (approximately 40 mL/day to FS and FST groups compared to 15 mL/day to C and CT) to maintain the isotonicity of body fluids. Accordingly, this partly entails that depletion of extracellular fluid volume is evidenced by increased haematocrit observed in fructose-salt load. However, the absence of sodium retention observed in association with a fructose/high-salt diet does not indicate that renal function was unaltered and does not favour a major implication of hemodynamic changes in this model. In the absence of reduced sodium excretory capability, continuous natriuresis would have accompanied the increase in arterial pressure until the pressure returned to control levels. In this regard, substantial capacity of the kidney for sodium excretion provides a compensatory system of gain-to-oppose processes, and interestingly, BP elevation was affected by Tempol. We suggest that there is a beneficial renal influence of Tempol on systemic hemodynamic effect in the present model. This is consistent with the fact that Tempol reduces BP by preventing an auto-regulatory adjustment of pre-glomerular vascular resistance as previously demonstrated^19^. Changes in serum blood urea nitrogen, urea, and creatinine concentrations may reflect alterations in glomerular filtration rate, which indicates abnormalities in urinalysis as the mechanism of altered renal homeostasis (abnormal volume regulation).

Additionally, several lines of evidence suggested that uric acid is responsible for the BP elevation and for the renal injury. Uric acid is one of the major metabolic end products of fructose and we found that co-treatment with a ROS scavenger normalized uric acid levels in FS rats. As reported earlier^20^, a high basal level of ROS is largely unfavorable for manifestation of the antioxidant properties of uric acid and increasing evidence suggests that it acts as a pro-oxidant once inside cells. Ejaz *et al*.^21^ have reported that uric acid stimulates production of ROS via its action on NADPH oxidase, followed by formation of ROS and lipid peroxidation. Furthermore, we revealed that another role of the antioxidant might be to protect against HDL from oxidative modifications. As triglycerides and cholesterol are transported predominantly by HDL in rats, we found that the effect of fructose-salt treatment decreasing the concentration of total cholesterol was due to a decrease of HDL. Superoxide has been reported to be a precursor for more chemically reactive oxidants with participation in the LDL oxidation by hydrolysing lipid peroxides^22^ and regulatory mechanisms of antioxidant enzymes prevent their restoration to a normal level, protecting the cell from the effects of ROS. In fact, there are possible routes by which cellular metabolism in this model may have been altered, which in turn may have accelerated oxidative stress; since the increase in oxidative stress may be due to the generation of ROS and/or the reduction of antioxidant protection^23^.

Our study hypothesized that decreasing 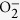 levels would have potential implications in the model studied. Several trials have been conducted to determine whether antioxidant treatment may reduce BP, but the results are conflicting^24^,^25^,^26^. Extracellular SOD, the first-line endogenous defense, is normally present in living tissues and converts 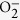 to hydrogen peroxide (H_2_O_2_) that is degraded to water and molecular oxygen by CAT^27^. However, although SOD and other antioxidants normally react with 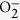, keeping them at very low levels, administration of antioxidant enzymes such as SOD, to prevent or treat cardiovascular damage^28^,^29^, is limited because they cannot permeate biological membranes and are therefore incapable of removing the intracellular 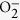 produced. SOD has limited membrane permeability and is unsatisfactory at preventing the adverse effects of 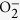 or reducing BP. Alternative agents with SOD-like activity have been investigated, and among these, Tempol demonstrates attenuation of hypertension^30^. Along this line, low activity of SOD renal was observed in FS rats and low levels of SOD are present in IR and in hypertension in humans studies and experimental animals^31^,^32^,^33^. We provided strong functional data to support involvement of a possibly inefficient antioxidant system causing hypertension. SBP and DBP were significantly increased in FS in comparison with those in control rats, but blocked by chronic administration of Tempol. In support of this possibility, the generation and action of the oxidative and antioxidant substances depends on the oxidation-reduction system, which shows that the imbalance between ROS production and the ability of the organism to deal with these reactive compounds can prevent or not the adverse effects arising from these changes. Moreover, a beneficial effect of Tempol treatment was associated with an anti-inflammatory effect along with a decrease in lymphocyte profile although accompanied with an increase in monocyte profile, which can lead to macrophage infiltration in the tissue.

In renal cells treated with fructose, salt and Tempol was observed a reduction in 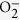 production. Hypertensive end organ damage is predominantly caused by inflammatory cells such as macrophages and lymphocytes infiltrating target organs. When these events occur in the vessel they predispose to progressive renal damage and ultimately renal insufficiency. Experimental study show that TNF-α enhances the local generation of ROS, enhancing albumin permeability through alteration of the glomerular capillary wall barrier^34^. Our results suggest an inflammatory effect as demonstrated by NF-κB activation to fructose-salt treatment in renal tissue, which is possible consider that increased ROS production should cause infiltration of macrophages and inflammation in renal tissue decreasing SOD activity. Importantly, our *in vitro* study revealed that Tempol reduced ROS production in renal tissue of fructose-salt treatment, and demonstrates a possible contribution of ROS via the NF-κB pathway, acting as a functional link in the BP-lowering effects of Tempol, which could be responsible for these processes fostering the activation of NF-κB. The elevated NF-κB expression and activation found in cultured HEK293 cells are in agreement with previous studies in renal model of hypertension^35^ and indeed, an increased expression of the nuclear transcription factor NF-κB will likely be involved to elicit the full biological response to high fructose and salt treatment.

In conclusion, the present findings suggest that fructose-fed rats subjected to salt overload show an adaptive response resulting in hypertension that can be counter-regulated by chronic treatment with a scavenger of 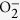. Notably, we found that enhanced salt intake might mediate the close association between hypertension, impaired glucose metabolism, and tissue ROS production serving as second messengers to NF-κB activation, which emphasizes a plausible role of NF-κB modulating the renal effects of hypertension.

## Materials and Methods

### Chemicals

Tempol from Sigma-Aldrich (St. Louis, MO, USA) and kits for biochemical analysis were purchased from Labtest Diagnóstica SA (Lagoa Santa, MG, Brazil). The specific antibodies against NFκB p65 was obtained from Santa Cruz Biotechnology, Inc. (Dallas). Secondary antibody and High-Capacity cDNA Archive kit and 2′,7′-dichlorodihydrofluorescein (H2-DCFDA) were purchased from Invitrogen (Saint Aubin, France). Other chemicals were of reagent grade.

### Animal Experiments

All animal experiments in this study were approved by the Institutional Animal Care and Use Committee of the Federal University of Ouro Preto and the methods were carried out in accordance with the approved guidelines. Adult Fischer male rats (Laboratory of Experimental Nutrition, School of Nutrition, Ouro Preto, Brazil) were used for the different experiments and were housed individually in wire-mesh cages throughout the study. The colony room was maintained at standard conditions of temperature, humidity, and dark-light cycle and the animals were provided with food and fluids *ad libitum*. The animals were divided into the following groups: control group (C; n = 11) with AIN93M diet, high-salt group (S; n = 12) provided with 8% w/w NaCl added to the diet, and high fructose group (F; n = 24) provided with 20% w/v fructose dissolved in the drinking water. After 10 weeks, the diet of half of the rats from the F group was switched to a high-salt one (8% w/w NaCl; FS group). Separate groups of rats were also kept on a diet with simultaneous intake of fructose solution and high-salt diet for 20 weeks. From week 13 onwards, Tempol was administered by gavage (10 mg/kg per day) until the end of 20 weeks in both groups (control, CT and fructose/salt, FST; n = 12 in each group). Placebo to C and FS groups received tap water for the same period (n = 12 in each group). Food, liquid, salt, and fructose intake were available for 10 days consecutives three weeks before the final of the experiment. After 20 weeks all animals of each experiment were placed under deep anesthesia with isoflurane and euthanized. Immediately afterwards the kidney was dissected and rinsed in ice-cold saline and stored at −80 °C until analyzed for antioxidant activity of SOD and CAT. Blood samples were collected for biochemical analyses.

### Blood pressure and heart rate recording

The tail cuff noninvasive method was used to measure arterial pressure. The rats were trained before for the measurement of SBP and HR taken at 0, 8, and 18 weeks of the protocol, using the Kent RTBP2000 series Rat Tail System (Kent Scientific), as described by Turbino-Ribeiro *et al*.^36^. In another series of experiments, SBP, DBP, and HR from the pulsatile pressure signal also were measured in rats of all groups at the 0, 8, and 18 weeks of diet, by digital tail plethysmography (Panlab, LE5001).

### Responses to an oral glucose load

Two weeks before the animals were euthanized, an oral glucose tolerance test was performed to confirm whether the rats on the fructose-enriched regimen had developed IR. The animals were fasted overnight and were only provided with water to drink. On the morning of the test, a glucose loading dose (2.5 g/kg) was administered directly into the stomachs of conscious rats through a fine gastric catheter and a drop of blood was taken from the tail vein to determine blood glucose levels at 0, 30, 60, and 120 min after glucose administration. Blood glucose levels were measured using a glucometer (AccuChek, Roche Diagnostics). The glucose area under the curve (AUC) was calculated using the trapezoidal rule and expressed as mg · dL-1 · min^−1^.

### Measurement of antioxidant enzymes

Kidney homogenates of each animal were prepared in a 50 mM potassium phosphate buffer (pH 7.4) and were divided into aliquots. Protein concentration was determined according to the method described by Lowry *et al*.^37^. The SOD activity was assayed by monitoring the autoxidation of pyrogallol according to the method described by Marklund and Marklund^38^. 1U of activity was defined as the amount that produced 50% inhibition of pyrogallol autoxidation under standard assay conditions. CAT activity was measured spectrophotometrically as the rate of the decomposition of H_2_O_2_, as described previously^39^. H_2_O_2_ decomposition was calculated kinetically using the molar extinction coefficient 39.41 · L · mol^−1^ cm^−1^. Enzyme activities were expressed as units per milligram of protein.

### Differential leucocyte count

A drop of blood was used to prepare blood smears and thus was stained with Panotic Fast, which use essential components dyes of Romanowsky, as described previousy^40^. Counts of 100 leukocytes were performed and the results were expressed as subtypes identified in lymphocyte, monocyte or polymorphonuclear (PMN) cells.

### Cell culture and incubation

Human embryonic kidney-293 (HEK293) cells in DMEM (Gibco) with 4.5 g/L D-glucose, and supplemented with 10% bovine growth serum (HyClone Laboratories, Logan, UT) were cultured in a humidified cell culture incubator maintained at 37 °C and supplied with 5% CO_2_. For the mechanistic studies, cells were plated into plaques (12 wells) and when in 60–70% confluent were treated with either 30 mM of L-fructose, 500 meq NaCl added to medium, and Tempol (50 mM) for 4 h. Cells were then either used to isolation of RNA and quantitative real-time PCR analysis. Expression of genes of cultured cells was evaluated by quantitative real-time PCR as previously described^41^. Briefly, RNAs extracted were reverse-transcribed into cDNA using the High-Capacity cDNA Archive kit. Real-time quantitative PCR (RT-qPCR) was then performed using the SYBR Green PCR Master Mix on an Applied Biosystems 7900HT Fast Real-Time PCR System (Applied Biosystem, Woolston, UK). Quantitative analysis of PCR data was conducted with the 2DDCt method using glyceraldehyde-3-phosphate dehydrogenase (GAPDH) Ct values for normalization. Melting analysis was conducted to validate the specificity of PCR products. The sequence of the primers used were TNF-α Forward CCA GGC GGT GCC TAT GTC TC, Reverse CAT CCA CTC CAG CTG CTC CT; SOD1 Forward TCG AGC AGA AGG AAA GTA ATG G, Reverse CTG GAT AGA GGA TTA AAG; and SOD2 Forward TGT CCA AAT CAG GAT CCA CTG, Reverse CAT TCT CCC AGT TGA TTA CAT. Each sample was analyzed in duplicate PCR reactions. To investigate the involvement of ROS in NF-κB activation was assessed by the following methods. (*1*) Intracellular ROS generation was detected using a H_2_-DCFDA fluorescent assay as previously described^42^. Cells were incubated with 2 μM H_2_-DCFDA for 4 h at 37 °C. The cells were then washed with cold phosphate buffered saline (PBS), and finally harvested by scraping into phosphate buffer (10 mM, pH 7.4)*/*methanol (vol*/*vol) containing Triton X-100 (0.1%). Fluorescence intensity of each sample was determined by spectrofluorimetry using excitation*/*emission wavelengths of 498*/*520 nm. (*2*) Cells were collected and lysed with Ripa Buffer containing 1% protease inhibitor cocktail and phosphatase inhibitor cocktail (Roche, Mannheim, Germany) on ice. Aliquots containing equivalent total protein content, quantified using the BCA™ Protein Assay, were separated by a 4–10% SDS-PAGE gel and then transferred into a nitrocellulose membrane, which was further incubated for 1 hour with 5%BSA in TBS containing 0.1% Tween 20, and probed overnight with an anti-NFκB p65. Finally, blots were incubated with a horseradish peroxidase-conjugated secondary Ab against rabbit at a 1:1000 dilution for 1 h at room temperature. Protein bands were revealed by enhanced chemiluminescence and quantified by densitometry with Fusion-Capt software (Vilber Lourmat, Fusion FX7, France). HSC70 was used to normalize protein loadings and quantification was performed with the BIO-1D software.

### Analysis of other variables

Blood glucose, triglycerides, total cholesterol, HDL, albumin, uric acid, urea, creatinine concentrations were measured using commercial kits from Labtest Diagnóstica according to the manufacturer’s instructions. Serum electrolytes (Na^+^, K^+^) were measured by flame-photometry.

### Statistical analysis

Data analyses were performed using GraphPad Prism version 5 (GraphPad, La Jolla, CA, USA). Normal distribution of continuous variables was tested using the Kolmogorov–Smirnov test. Data in each group were compared by analysis of variance or Kruskal-Wallis tests, followed by the Dunnett’s and Dunn’s, respectively, while unpaired Student *t* test was used in the case of a simple comparison. Differences were considered significant at values of *P* < *0.05*.

## Additional Information

**How to cite this article**: Dornas, W. C. *et al*. Oxidative stress causes hypertension and activation of nuclear factor-κB after high-fructose and salt treatments. *Sci. Rep.* **7**, 46051; doi: 10.1038/srep46051 (2017).

**Publisher's note:** Springer Nature remains neutral with regard to jurisdictional claims in published maps and institutional affiliations.

## Figures and Tables

**Table 1:** Effects of high fructose and salt plus Tempol on blood parameters.

	C	CT	FS	FST
Glucose (mmol/L)	7.54 ± 0.2	6.57 ± 0.2	9.99 ± 0.6*	8.84 ± 0.6
Triglyceride (mmol/L)	1.88 ± 0.2	1.97 ± 0.1	3.12 ± 0.2*	2.64 ± 0.2
Total cholesterol (mmol/L)	2.8 ± 0.2	3.29 ± 0.1	1.77 ± 0.2*	2.1 ± 0.4*^,†^
HDL cholesterol (mmol/L)	2.14 ± 0.2	2.28 ± 0.3	1.12 ± 0.1*	1.62 ± 0.3*^,†^
Albumin (g/dL)	3.31 ± 0.3	3.36 ± 0.2	3.6 ± 0.2	3.4 ± 0.1
Urea (mg/dL)	36.9 ± 3.7	35.2 ± 3.3	45.3 ± 5.7*	38.2 ± 7.3^†^
Creatinine (mg/dL)	0.97 ± 0.05	1.04 ± 0.07	1.3 ± 0.1*	1.07 ± 0.09^†^
Uric acid (mg/dL)	1.78 ± 0.9	1.41 ± 0.6	2.38 ± 0.8*	1.62 ± 0.4^†^
Sodium (meq/L)	142.0 ± 1.7	139.3 ± 1.2	135.5 ± 1.2	135.0 ± 1.7
Potassium (meq/L)	4.53 ± 0.08	4.66 ± 0.12	4.66 ± 0.09	4.86 ± 0.08
Haematocrit (%)	27.73 ± 2.9	31.0 ± 2.0	31.92 ± 2.9*	39.9 ± 1.4*

Abbreviations: HDL, high density lipoprotein; C, control group; CT, control group + Tempol.

FS, high fructose and salt group; FST, high fructose and salt group + Tempol.

^*^P < 0.05versus C.

^†^*P* < 0.05 versus FS.
